# Appearance and management of COVID-19 laryngo-tracheitis: two case reports

**DOI:** 10.12688/f1000research.23204.2

**Published:** 2020-08-20

**Authors:** Charles Matthew Oliver, Marta Campbell, Oma Dulan, Nick Hamilton, Martin Birchall

**Affiliations:** 1 Departments of Anaesthesia and Intensive Care Medicine, Royal Free Hampstead NHS Trust Hospital, London, nw3 2qg, UK; 2 Division of Surgery and Interventional Science, University College London, London, W1W 7TS, UK; 3 University College London Hospitals NHS Trust, London, NW1 2BU, UK; 4 Ear Institute, University College London, London, WC1X 8EE, UK; 5 NIHR Biomedical Research Centre, University College London Hospitals, London, UK

**Keywords:** COVID, SARS-CoV-19, Intensive care, Airway management

## Abstract

We present two cases of coronavirus disease 2019 (COVID-19)-related laryngotracheitis in good-prognosis, ventilated patients who had failed extubation. As the pandemic continues to unfold across the globe and better management of those with respiratory failure develops, this may be an increasingly common scenario. Close ENT-intensivist liaison, meticulous team preparation, early consideration of rigid endoscopy and prospective data collection and case sharing are recommended.

## Introduction

Coronavirus disease 2019 (COVID-19) infection, caused by the SARS-CoV-2 virus, is presently declared a global pandemic responsible for 571,678 reported cases and 26,494 deaths at the time of writing. Initial symptoms commonly include fever and cough with a delayed onset of progressive breathlessness ^1^. In the largest Chinese cohort of 1099 patients, mechanical ventilation was required in 2.3% ^2^, although figures from Lombardy in Italy show higher rates and ICU bed provision has had to double in the space of six weeks ^3^. Pressure on intensive care systems is now so great internationally that an understanding of the processes for delayed tracheal extubation is very important. We describe two patients whose extubation and discharge were delayed due to florid COVID-19-related laryngo-tracheitis causing upper airway obstruction.

## Case report 1

**Figure 1.**
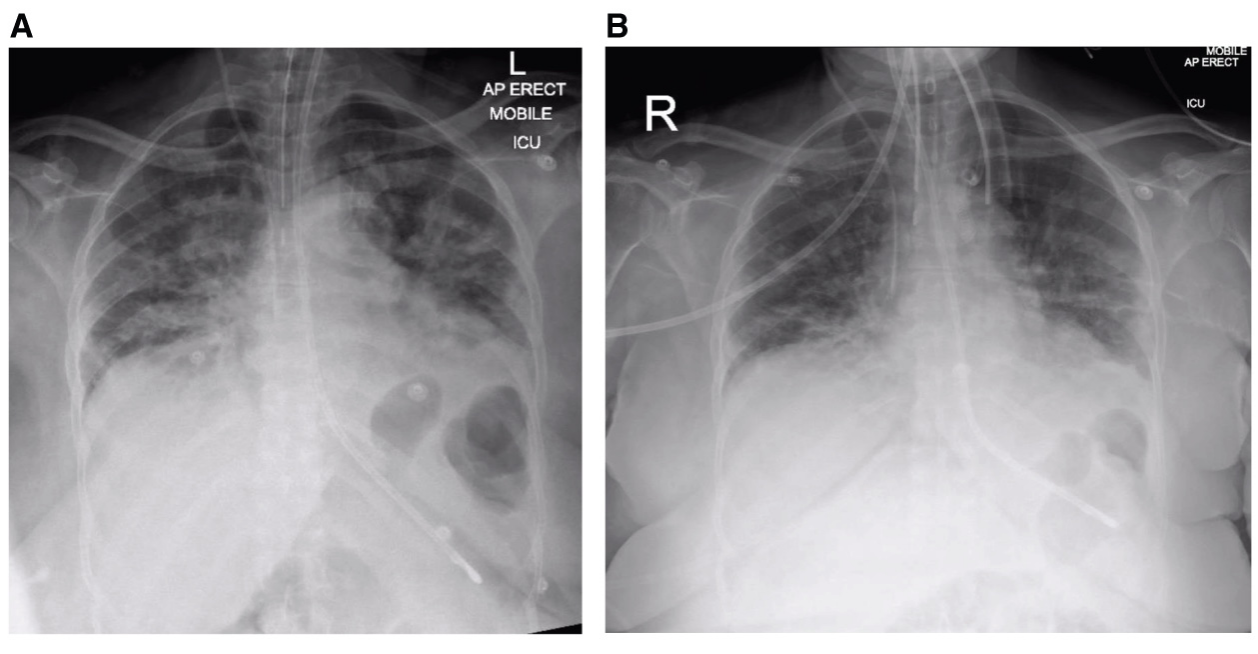
Case 1 radiographs. ( **A**) Post-intubation plain chest radiograph, on ICU on day 5 post-onset of symptoms. ( **B**) Plain chest radiograph on day 10 post-onset of symptoms.

A 69-year-old female, non-smoker with a background history of hypertension (controlled by amlodipine 5 mg once daily) presented to the emergency department with a three-day history of pyrexia and tachycardia. Admission chest X-ray (CXR) showed bilateral pulmonary infiltrates. On day 5 after onset of symptoms, she was transferred to the intensive care unit where she required tracheal intubation and invasive ventilation for worsening type 1 respiratory failure. An 8-mm internal diameter endotracheal tube (ETT, Portex, Hythe, UK) was sited on first attempt with video-laryngoscopy, secured at 22 cm at the lips, with tip position subsequently confirmed on CXR ( Figure 1A) ^4^. Laryngoscopy view was grade 1 ^5^, and no pathology was recorded.

With reducing levels of ventilatory support requirement (spontaneous effort, FiO _2_ 0.3, pressure support (PS) 5 cm H _2_O, positive end expiratory pressure (PEEP) 5 cm H _2_O, extubation was attempted five days later (day 10), but was unsuccessful due to excessive resistance to egress of the ETT. When repeat video-laryngoscopy suggested laryngeal oedema, 6.6 mg three times daily dexamethasone was commenced. Repeat CXR demonstrated no causative pathology ( Figure 1B). Two further attempts at extubation over successive days again failed, characterised by lack of audible leak after cuff deflation and almost complete immobility of the tube on reasonable traction.

Following careful planning between clinicians and managers across two sites, on day 19 the patient was transferred to an operating theatre for laryngoscopy and bronchoscopy. Ventilatory parameters were unchanged, she required no additional organ support and only minimal sedation (propofol and fentanyl) was required to ensure ETT tolerance. On the day of surgery, two iterative team briefs were conducted, during which all team members were asked to contribute questions and suggestions; a plan was agreed with all potential anticipated events and adverse events considered, along with their mitigation, and equipment located.

All team applied full personal protective equipment (PPE), comprising: FFP3 mask (fit-checked and leak-tested by trained testers), visor, apron, gown, two pairs of gloves and PPE footwear. Communication between in-theatre staff (‘COVID-19 team’) and external support staff (nurse and ODP, non-COVID team) was established with two-way radios. The patient was transferred onto an anaesthetic ventilator, neuromuscular blockade administered (rocuronium 50 mg) and ventilated on mandatory mode, FiO _2_ 1.0. General anaesthesia was maintained with propofol and fentanyl infusions and further rocuronium boluses were administered. A tracheostomy set was prepared with size 6 and 7 cuffed non-fenestrated tubes (Portex, Hythe, UK) tested and pre-loaded with introducers in case of upper airway obstruction.

Laryngoscopy was performed using a combination of adult Lindholm and Dedo laryngoscopes (Karl Storz, Jena, Germany), to visualise the supraglottis and glottis respectively. Laryngoscopes were placed on suspension without the need for counter-pressure and imaging performed using a 0 ^o^ Hopkins’ rod telescope and camera system (Karl Storz, Jena, Germany). Bronchoscopy via a T-piece port attached to the ETT was performed using a disposable bronchoscope (Broncho Slim, Ambu, Ballerup, Denmark). This showed that the lower trachea, main and lobar bronchi were normal with no obvious mucosal oedema, excessive secretions or ulceration.

**Figure 2.**
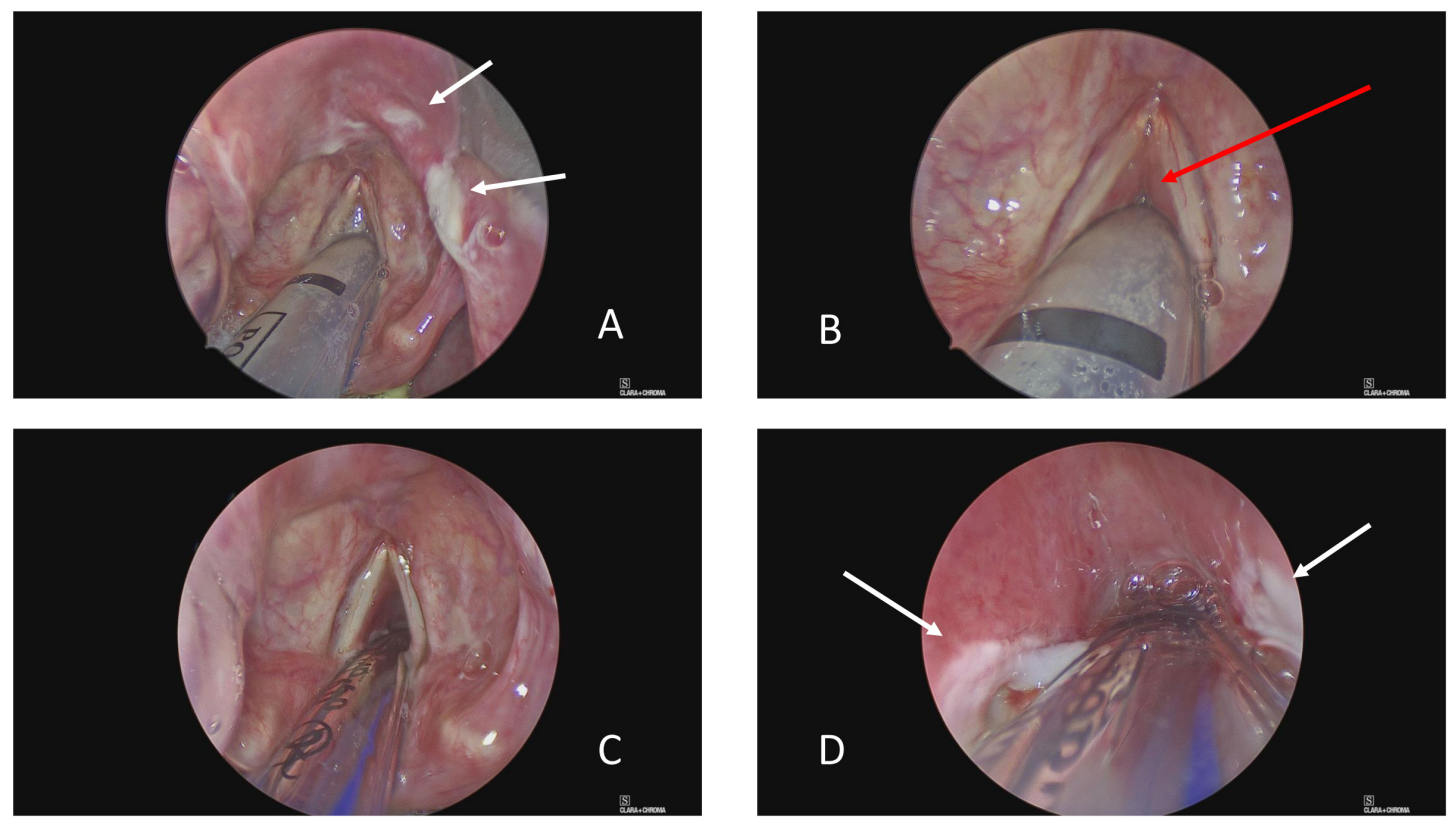
Case 1 glottis images. ( **A**) View of supraglottis showing ulcerated epiglottis. ( **B**) Glottis showing relative sparing of vocal cords and false cords, but profound subglottic oedema. ( **C**) Following change to size 6 endotracheal tube, there is some anterior glottic airway. ( **D**) However, the subglottis is also ulcerated and oedematous mucosa prevents rigid bronchoscopy (0 ^o ^Hopkins’ rod) beyond the third tracheal ring. White arrows indicate areas of ulceration and red arrow subglottic oedema.

The epiglottis was inflamed with shallow, irregular, ulcers ( Figure 2A). A sample of the ulcerated area was sent for microbiology testing. The rest of the supraglottis and superior surface of the vocal cords were spared, whilst profound oedema encased the ETT from cord level downwards ( Figure 2B). It was not possible to pass the Hopkins’ rod past cord level. Adrenaline 1:10,000-soaked neurosurgical patties were packed around the tube in the glottic and subglottic area for 15 minutes to try and reduce swelling and risk of bleeding, and then removed using microlaryngeal instruments. Following pre-oxygenation and apnoea, a paediatric endotracheal tube bougie (10 ch × 600 mm, P3 Medical Ltd) was introduced through the ETT, the ETT was removed atraumatically with steady traction and a size 6 ETT then “railroaded” over the bougie (under direct rigid laryngoscopic) vision to replace it. Ventilation was recommenced without incident. Hopkins’ rod examination was now possible through the newly patent anterior glottis ( Figure 2C), but only as far as the fourth tracheal ring due to upper tracheal and subglottic oedema. Ulcers were present bilaterally in the subglottis ( Figure 2D). Depomedrone (40 mg/ml, 0.3 ml per side) was injected into the subglottis using a modified butterfly needle.

The theatre team “doffed” (removed protective clothing) in a dedicated anteroom, immediately adjacent to the operating theatre and showered. A debrief was then held where all learnings, thoughts and feelings were recorded. The values of planning, repetition of plans, risk anticipation and effective communication and egalitarian team-work were highlighted. Problems identified were the difficulties in communicating verbally between theatre staff and between those inside and outside theatre due to protective clothing and protocols, and the time and expertise required to prepare adequately and safely for a high-risk COVID-19 airway case.

### Outcome and follow up

The patient was returned to ICU following the procedure, where supportive treatment and systemic corticosteroid treatment was continued. On day 23, following confirmation of ‘cuff leak’, she was successfully extubated. On day 25 she was stepped down to a level 1 bed.

## Case report 2

**Figure 3.**
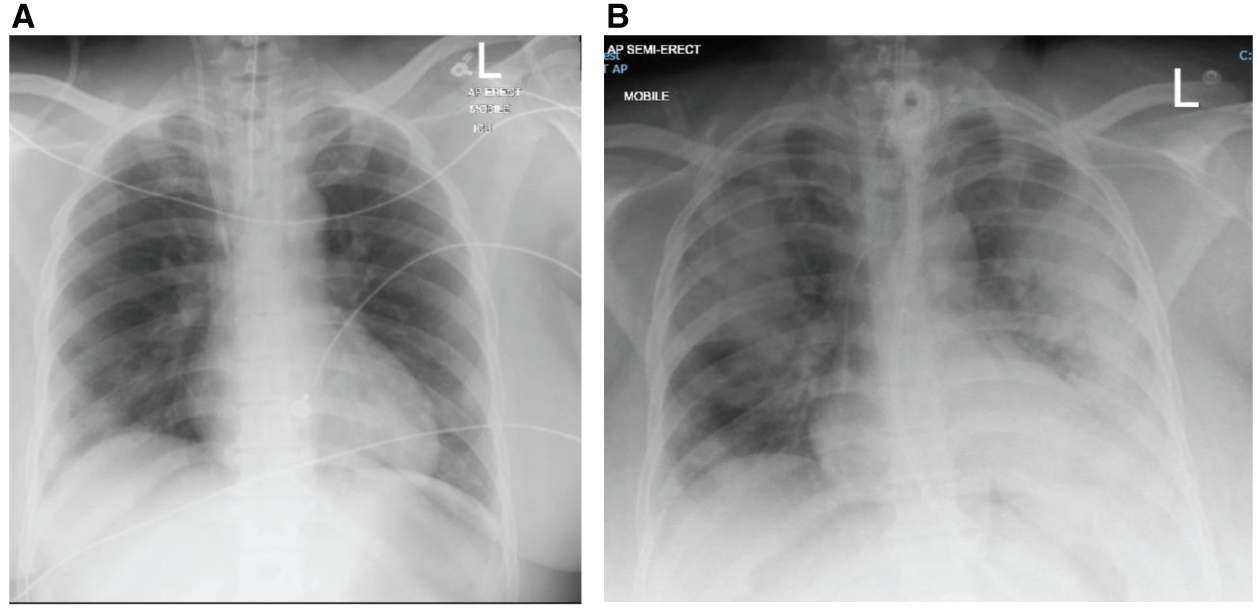
Case 2 radiographs. ( **A**) Post-intubation plain chest radiograph, on day 1 of hospital admission. ( **B**) Day 5 following re-intubation.

A 45-year-old female with poorly controlled, insulin-dependent diabetes mellitus (with retinopathy), hypothyroidism and central adiposity presented to our emergency department in extremis, in diabetic ketoacidosis, severely dehydrated and agitated following two days of cough and anorexia. The cough was non-productive. Arterial blood gas results included pH 6.91, Base -26, blood sugar level was high (unrecordable) and ketones were elevated at 5 mmol/L. A size 7.0-mm cuffed oral endotracheal tube was chosen to permit invasive ventilation and bronchoscopy if required; Cormac & Lehane view was Grade 2 and the tube was fixed at 23 cm from the lips. Initial CXR, Figure 3a. Medications on presentation were metformin 1 g twice daily, Lantus & Novorapid (variable doses) and levothyroxine 100 µg once daily.

She was transferred to an isolation room on the main intensive care unit, started on a fixed rate intravenous insulin infusion (0.1 units/kg/h), fluid resuscitated and started on ceftriaxone (per protocol) and clarithromycin.

On day 5 of admission, the ETT was removed in a trial of extubation. She was stridulous, not improving with nebulised adrenaline and intravenous corticosteroids, and progressively developed increased work of breathing. She was re-intubated (again size 7) several hours later and started on regular dexamethasone 6.6mg TDS. Subsequent CXR, Figure 3b.

**Figure 4.**
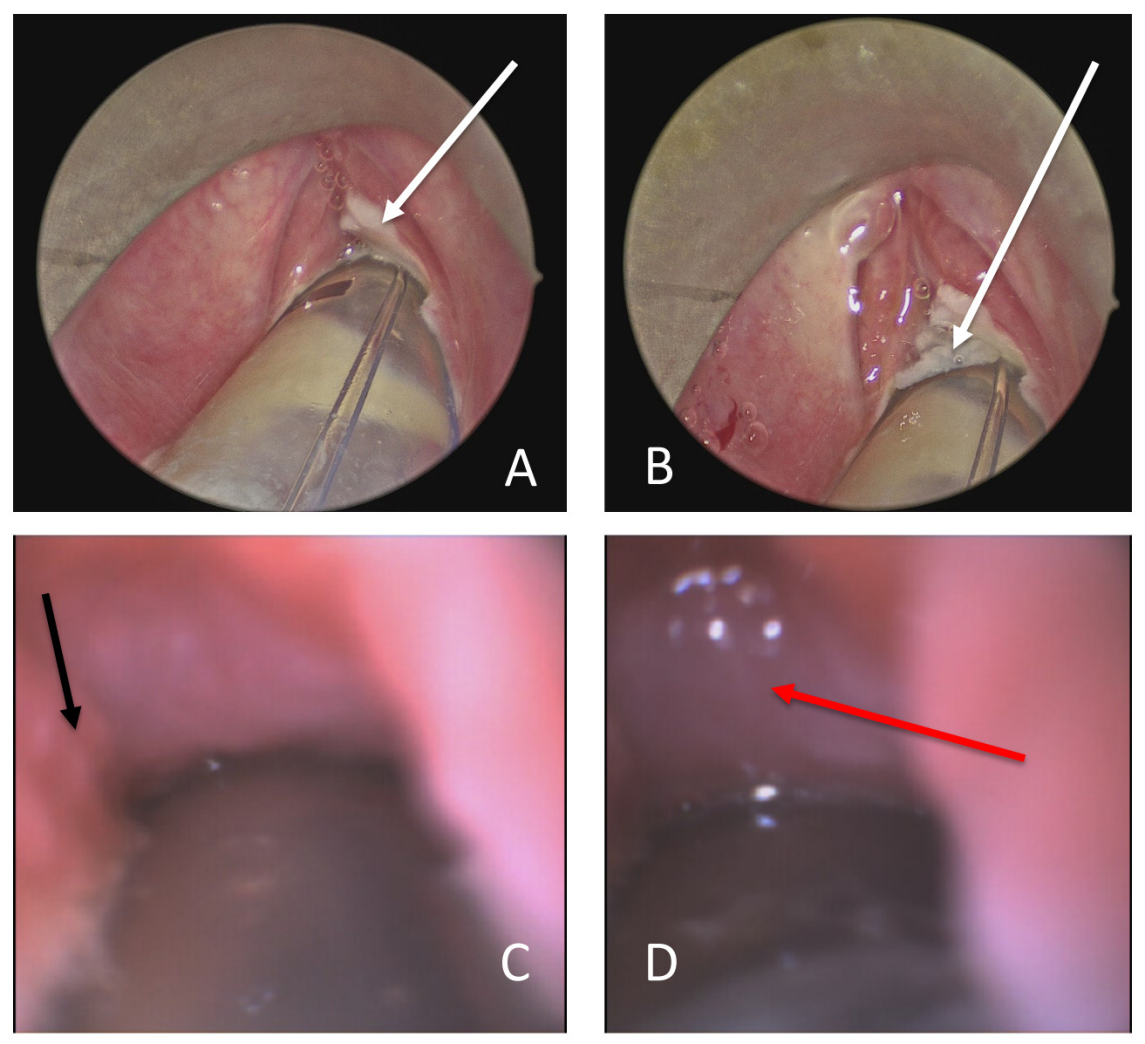
Case 2 glottis images. ( **A**) View of supraglottis showing ulcerated glottis. ( **B**) Glottis showing sparing of false cords, but profound glottic oedema and glottic and subglottic ulceration. ( **C**) Flexible bronchoscopy via the anterior commissure shows subglottic oedema and granulation tissue (black arrow). ( **D**) Oedematous mucosa prevents flexible bronchoscopy beyond the third tracheal ring. White arrows indicate areas of ulceration, black arrow granulation tissue and red arrow tracheal oedema.

On day 13 she remained suitable for extubation by pulmonary and other measures, but no cuff leak was present when assessed. On day 15 she underwent a surgical tracheostomy preceded by microlaryngoscopy and bronchoscopy. At microlaryngoscopy there was profound oedema in the glottis and subglottis ( Figure 4). Passage of a disposable fine-bore bronchoscope (Broncho Slim, Ambu, Ballerup, Denmark) through the anterior commissure revealed extensive tracheal oedema with some granulation tissue and ulceration in the subglottis. It was deemed impossible to extubate due to the swelling and so tracheostomy was performed according to the UCLH COVID19 tracheostomy protocol. In brief, through a small collar incision, the trachea was approached using only clips and ties to reduce the risk of inhaled virus-rich “plume” from diathermy. After pre-oxygenation, the ETT was advanced beyond the site of the tracheostomy with the balloon fully inflated and ventilation suspended. A window was created revealing again oedematous mucosa and the endotracheal tube withdrawn under direct vision until the tip was just higher than the window. A size 7 tracheostomy tube (Blueline Ultra, PORTEX, Hythe, Kent) was placed. A pre-loaded closed suction and ventilation extension, with a viral filter, was attached, the cuff inflated, and ventilation recommenced. The tube was sewn in place at all four poles and ties added. Post-operatively she steadily improved and, on day 22, tracheostomy wean was progressing well. By day 7 after surgery, intraoperative samples had grown no pathological bacteria.

## Discussion

Viral upper airway infection may present as a spectrum ranging from dysphonia to fulminant airway compromise, representing oedema, inflammation and ulceration. In a literature review, we identified case reports of clinically significant epiglottitis, laryngitis and tracheitis associated with less commonly encountered viral pathogens (HSV, HZV and HIV) ^6– 8^. Anecdotally, glottic oedema has been seen as a presenting feature of COVID-19 in an infant (C. Frauenfelder, Great Ormond Street Hospital for Children, personal communication 29th March 2020). However, upper airway involvement has however yet to be formally reported in coronavirus infection in humans to our knowledge.

The coronavirus enters cells by binding to the angiotensin converting enzyme 2 (ACE2) receptor which is found on the apical surface of differentiated ciliated respiratory epithelia ^9– 11^. This cell type is particularly dense in airway epithelial cells, hence the severity of COVID-19 disease in lungs and distal airways. However, the adult glottic and supraglottic larynx has variable areas of ciliated respiratory cells ^12^, which may explain why only parts of the supraglottis were affected whilst the subglottis and trachea were profoundly oedematous. In chickens, coronavirus infection is associated with laryngotracheitis ^13^, but this condition has not previously been described in primates or humans.

These cases highlight the need for close interdisciplinary working and communication in the management of airway complications of COVID-19 infection. Here, careful joint planning between anaesthetists and ENT (laryngology specialist) surgeons was critical. We recommend daily laryngology/head and neck surgeon meetings with ICU staff during such pandemics ideally through the use of video conferencing software to limit potential spread between healthcare workers. Meetings should discuss issues on a case-by-case basis with written protocols designed to carefully balance risk and benefit of, especially, tracheostomy. In the first case presented, such dialogue obviated the need for tracheostomy.

Full PPE and COVID-19 protocols require a new approach to theatre communication. Task-specific equipment, such as disposable ear-pieces or throat microphones, might be developed where they do not compromise mask seals. Communication protocols, such as those used by airlines and the military, may be introduced.

The key findings in the present cases were ulceration of the epiglottis and subglottis and profound oedema and granulations in the subglottis and upper trachea. These changes were observed despite resolution of clinical, radiological and bronchoscopic characteristics of COVID-19 respiratory disease and clinical improvement based on reduction in oxygen and ventilation needs. The relatively late and prolonged response of this part of the airway may be idiosyncratic and the true incidence and demographics of COVID-19 laryngotracheitis (C19LT) will only be understood by prospective national/multinational case and data collection.

Prior to the theatre procedure, we used systemic steroids to try and reduce upper airway oedema. In the present cases, its use did not avoid the ultimate need to resort to rigid endoscopy and experience with previous SARS epidemics suggest systemic steroids may increase viral shedding ^14^. We hypothesise that early consideration of such endoscopy, especially in “good prognosis” patients, may be indicated rather than a trial of steroids. Likewise, it could be argued that an intra-laryngeal injection of depot steroids in the first case may slow rather than assist local resolution of oedema. Again, prospective data collection is required to answer these questions.

Tracheostomy represents the third highest risk of COVID-19 transmission to staff after ETT intubation and non-invasive ventilation ^15^. Reports from Hong Kong, which experienced high levels of SARS-1 and SARS-2 cases, highlights the need to delay or avoid tracheostomies in this group of patients where clinically possible ^16– 18^. Whether tracheostomy can expedite extubation and free up ventilator capacity during the COVID-19 pandemic is not yet established and should be the focus of research activity. The narrowing, oedema and ulceration of the trachea in exactly the location where a tracheostomy, either open or percutaneous, would be performed suggests that such procedures may be more hazardous and present more post-operative problems than in those without such oedema. In selected cases, rigid endoscopy may be useful in defining the pathology.

### Learning points

- Coronavirus may cause symptomatic inflammation of the larynx as well as the trachea, bronchi and lungs, resulting in difficulties in both tracheal intubation and extubation.
- A distinct condition of COVID-19-related laryngotracheitis may exist. This may make siting of tracheostomy tubes even more problematic due to narrowing of the airway, thickening of mucosa and increase in local secretions.
- Early consideration of this diagnosis and endoscopy may be considered.
- Tracheal intubation and extubation of the patient with COVID-19 may be a high-risk procedure for staff, irrespective of the clinical severity of disease. Where possible, Aerosol generating procedures (AGP) should be performed in a negative pressure room with > 12 air changes per hour whenever possible.
- Tracheal intubation and extubation of the patient with COVID-19 may be a high-risk procedure for staff, irrespective of the clinical severity of disease.
- Meticulous planning with the full theatre team is required before embarking on all airway procedures in COVID19 infected patients.
- Communication issues due to the wearing of PPE in operating theatres require novel solutions.

## Data availability

All data underlying the results are available as part of the article and no additional source data are required.

## Consent

Written informed consent for publication of their clinical details and clinical images was obtained from the patients.
