# Supplementary material for: Appearance and management of COVID-19 laryngo-tracheitis: two case reports
Source: F1000Res. 2020 Aug 20;9:310. Originally published 2020 Apr 29. [Version 2] doi: 10.12688/f1000research.23204.2 (PMC7364183; doi:10.12688/f1000research.23204.2)
Supplement: Peer review history

# Peer review: Reviewer response for version 1

**DOI:** 10.5256/f1000research.25616.r65373

**Published:** 2020-07-15

**Authors:** Jean Paul Marie

**Affiliations:** 1 Unit of Otorhinolaryngology—Department of Surgery and Physiology, Faculty of Medicine, University of Porto, Porto, Portugal

**Introduction :**

 "We describe a patient whose extubation and discharge were delayed due to a florid COVID-19-related laryngo-tracheitis causing upper airway obstruction."

 The title of the paper is now 2 patients. Has to be corrected.

**Case report 1**

 “An 8-mm external diameter endotracheal tube (ETT, Portex, Hythe, UK)"

 as it a n° 8 portex tube. Are you sure that external diameter is 8 mm?

**Case report 2**

 OK.

**Discussion**

 Well built.

Are enough details provided of any physical examination and diagnostic tests, treatment given and outcomes?

Yes

Is the background of the cases’ history and progression described in sufficient detail?

Yes

Is sufficient discussion included of the importance of the findings and their relevance to future understanding of disease processes, diagnosis or treatment?

Yes

Is the conclusion balanced and justified on the basis of the findings?

Yes

Reviewer Expertise:

I am an ENT, specialized in airway and neurosciences

I confirm that I have read this submission and believe that I have an appropriate level of expertise to confirm that it is of an acceptable scientific standard.

# Peer review: Reviewer response for version 1

**DOI:** 10.5256/f1000research.25616.r63333

**Published:** 2020-05-15

**Authors:** Jeyasakthy Saniasiaya

**Affiliations:** 1 Department of Otorhinolaryngology, Hospital Tuanku Ja'afar, Seremban, Malaysia

Overall, this is a clear, well-written manuscript. Adequate information especially on patient's presentation as well as approach was described.

 The introduction is relevant. Adequate information on previous study findings on viral laryngotracheitis  is mentioned for readers to follow. Also, it is interesting that step-by-step airway assessment was provided for readers which is especially prudent during this period. 

 However, the authors need to mention on why biopsy was not performed in this case. This manuscript definitely adds value to the current COVID-19 pandemic.

Are enough details provided of any physical examination and diagnostic tests, treatment given and outcomes?

Yes

Is the background of the cases’ history and progression described in sufficient detail?

Yes

Is sufficient discussion included of the importance of the findings and their relevance to future understanding of disease processes, diagnosis or treatment?

Yes

Is the conclusion balanced and justified on the basis of the findings?

Yes

Reviewer Expertise:

Otorhinolaryngology

I confirm that I have read this submission and believe that I have an appropriate level of expertise to confirm that it is of an acceptable scientific standard.

# Response

**Published:** 2020-08-04

**Authors:** Charles Matthew (Matt) Oliver

**Affiliations:** UCL Centre for Anaesthesia Critical Care & Pain Medicine, University College London, UK

Thank you for your reviews, which we have used to improve the quality of this manuscript.

Regarding biopsies: We wanted to reduce the potential for transmission as much as possible. In retrospect, we would have taken a biopsy if we knew what we know now and had updated systems in place.
